# Redo Axillary Artery Cannulation in Reoperative Cardiac Surgery: Outcomes and Technique

**DOI:** 10.1016/j.atssr.2026.01.020

**Published:** 2026-03-04

**Authors:** Lauren V. Huckaby, Andrew M. Vekstein, Peter S. Downey, Julie Doberne, Jovan Markovic, Jeffrey G. Gaca, G. Chad Hughes

**Affiliations:** 1 Division of Thoracic and Cardiovascular Surgery, Department of Surgery, Duke University Medical Center, Durham, North Carolina; 2 Division of Cardiac Surgery, Department of Surgery, UCLA Health, Los Angeles, California; 3 Division of Cardiothoracic Surgery, Department of Surgery, Oregon Health & Science University, Portland, Oregon

## Abstract

**Background:**

Reoperative sternotomy often necessitates peripheral arterial cannulation to initiate systemic or cerebral perfusion. In patients with prior right axillary cannulation, the site can be reused by anastomosing a new Dacron graft at, proximal to, or distal to the original anastomosis. Axillary cannulation provides reliable systemic perfusion while avoiding the embolic risks of femoral access and technical challenges of central cannulation in the redo setting. However, outcomes after redo axillary cannulation are sparsely reported, and the safety of this approach is not well defined.

**Methods:**

We retrospectively reviewed 55 consecutive patients (2008-2025) who underwent redo axillary cannulation for cardiopulmonary bypass during complex adult cardiac surgery at a single high-volume aortic referral center. Thirty-day/in-hospital outcomes were recorded, and operative technique was described. Redo cannulation involved anastomosis of a Dacron side-arm graft at the prior site or immediately adjacent along the axillary artery.

**Results:**

In 55 patients (mean age, 58.9 years; 72.7% male), redo axillary cannulation was performed a median of 4.6 years after the original operation. The most common indications for reoperation were aneurysm (60.0%) and structural valve degeneration (23.6%). Thirty-day mortality was 1.8%, and perioperative stroke occurred in 3.6% (n = 2). Ipsilateral upper extremity neurovascular complications were uncommon (3.6%), and no wound complications were observed.

**Conclusions:**

Redo axillary cannulation is technically feasible and safe, with low rates of stroke, neurovascular injury, and no wound complications in this series. Prior axillary cannulation should not preclude reuse of the artery, offering a reliable alternative when central or femoral cannulation is undesirable.

**In Short**

▪ Redo axillary cannulation is a safe and technically feasible method for establishing cardiopulmonary bypass during complex reoperations, with low perioperative morbidity in a consecutive series of 55 patients.
▪ Prior axillary use should not preclude reuse of the artery in reoperative sternotomy, particularly when central cannulation is not possible, and this approach offers a reliable alternative that avoids the retrograde embolic risks of femoral access.

The right axillary artery is frequently used for peripheral cannulation for systemic perfusion during cardiopulmonary bypass (CPB) as well as for antegrade cerebral perfusion.^1^, ^2^, ^3^ Both direct cannulation and cannulation through a side-branch graft have been described, with the latter associated with reduced rates of stroke and neurovascular complications.^4^ In patients undergoing reoperative sternotomy, particularly those with aortic disease, repeated axillary artery dissection and cannulation may be required. In many cases, a side-branch graft is retained, serving as both a landmark for reexposure and a conduit for redo anastomosis. Although axillary cannulation has been shown to reduce rates of stroke compared with femoral access, few studies detail the technical approach or outcomes of redo axillary cannulation in patients with prior axillary use.^5^ We therefore reviewed our institutional experience with redo axillary cannulation, describing both operative technique and perioperative outcomes.

## Patients and Methods

We conducted a retrospective review of consecutive patients undergoing redo axillary artery cannulation for CPB at our institution between 2008 and 2025. Patients who underwent redo axillary access solely for endovascular intervention (n = 5) were excluded. Our operative approach to redo axillary dissection and cannulation technique is described in detail later. Upper extremity complications were defined as any neurologic or vascular events requiring surgical intervention, initiation of new therapy (eg, anticoagulation), or additional outpatient rehabilitation. Wound complications were defined as any wound breakdown or infection requiring surgical or inpatient management or initiation of antibiotics.

## Results

### Preoperative Evaluation

In patients with prior axillary cannulation, a focused history should be obtained to assess for sequelae, such as pain or neurologic changes, or any perioperative events, such as infection or reoperation. The operative report should be reviewed for details of the conduct of the initial approach, including whether the artery was cannulated directly or through a side-branch graft. On physical examination, the location of the prior incision should be noted, along with a focused neurovascular examination of the extremity with bilateral radial artery pulse comparison, and the findings documented. Axillary incisions are typically lateral in the deltopectoral groove or more medial in the infraclavicular fossa. Preoperative cross-sectional imaging with contrast-enhanced computed tomography angiography should be performed in all cases involving redo sternotomy.^6^ The study should be reviewed to assess the size of the axillary artery and any evidence of postprocedure stenosis as well as the presence of any residual clips or graft material, which can serve as helpful landmarks during operative reexposure as detailed here.

### Operative Technique

**Figure 1:**
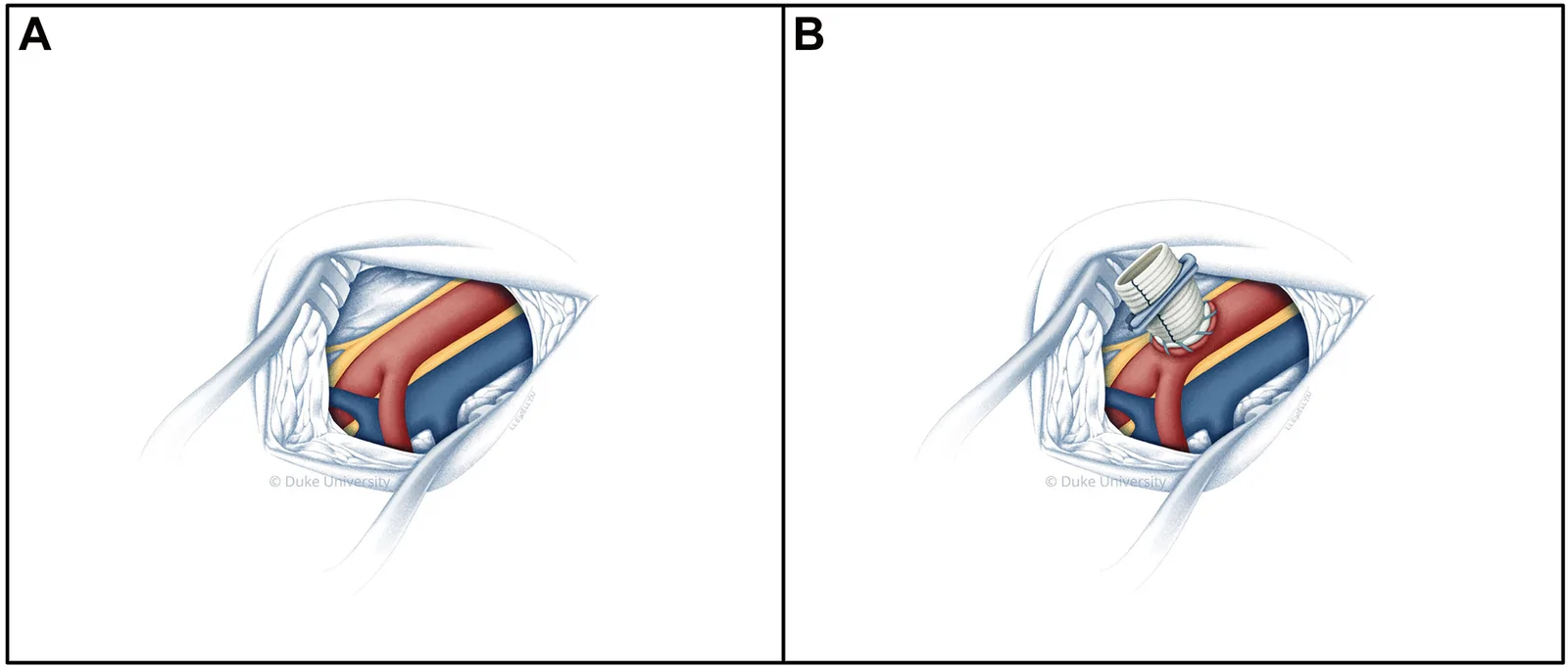
(A) Anatomy of the right axillary artery, demonstrating the proximity of the artery and axillary vein to the brachial plexus during the surgical exposure. (B) Prior cannulation of the right axillary artery with a retained Dacron graft stump, which can serve as a palpable landmark during redo surgery.

**Figure 2:**
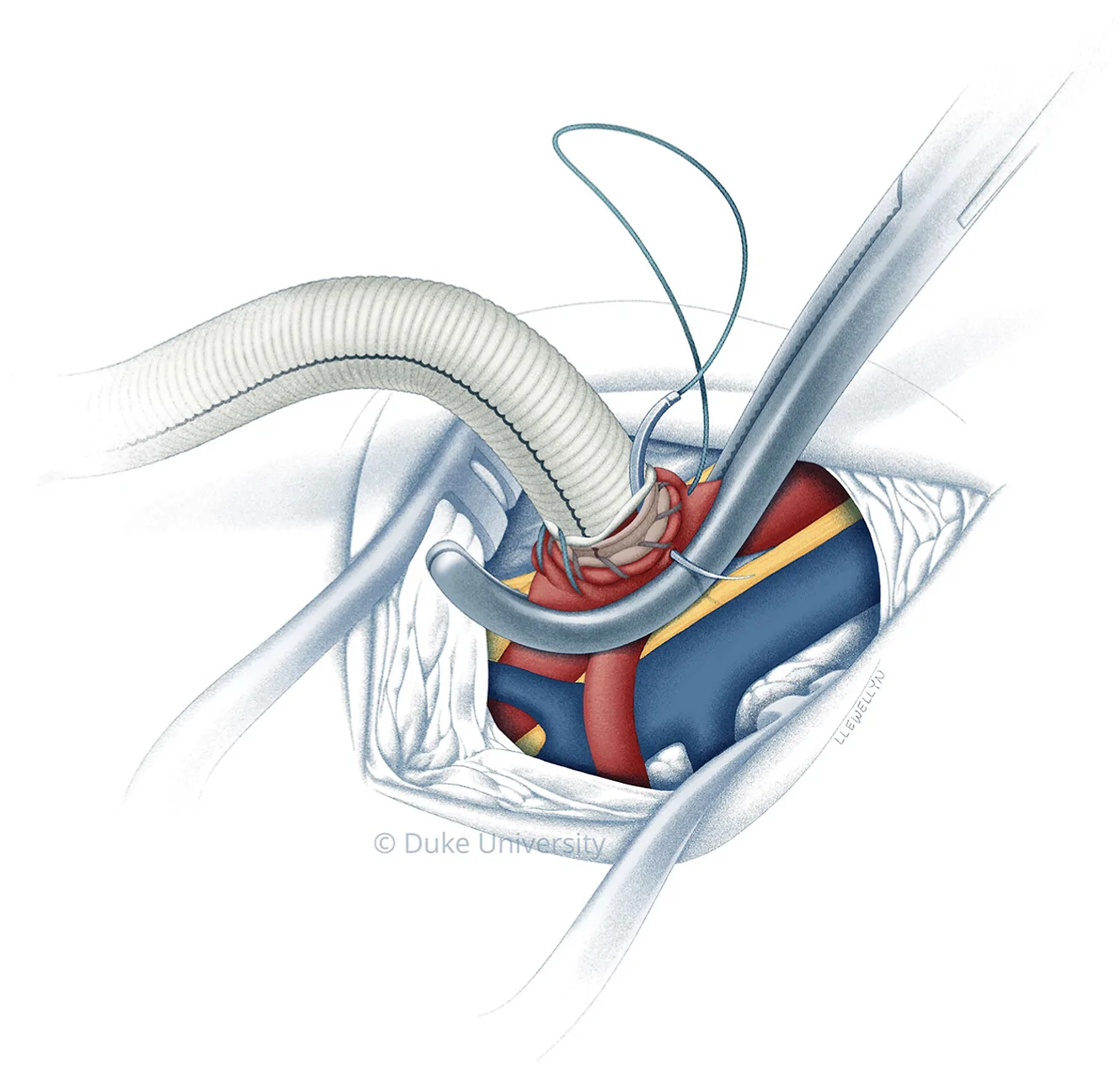
Redo cannulation at the prior anastomotic site. The residual Dacron cuff is trimmed short and a new graft anastomosed to the prior site, with the bites of the suture line incorporating both the remaining Dacron graft stump and approximately 1 to 2 mm of native artery below the stump to improve hemostasis. Whereas a Satinsky clamp (shown) is most commonly used in the primary setting, we prefer to use 2 profunda clamps (1 proximal and 1 distal) for redo cannulation.

**Figure 3:**
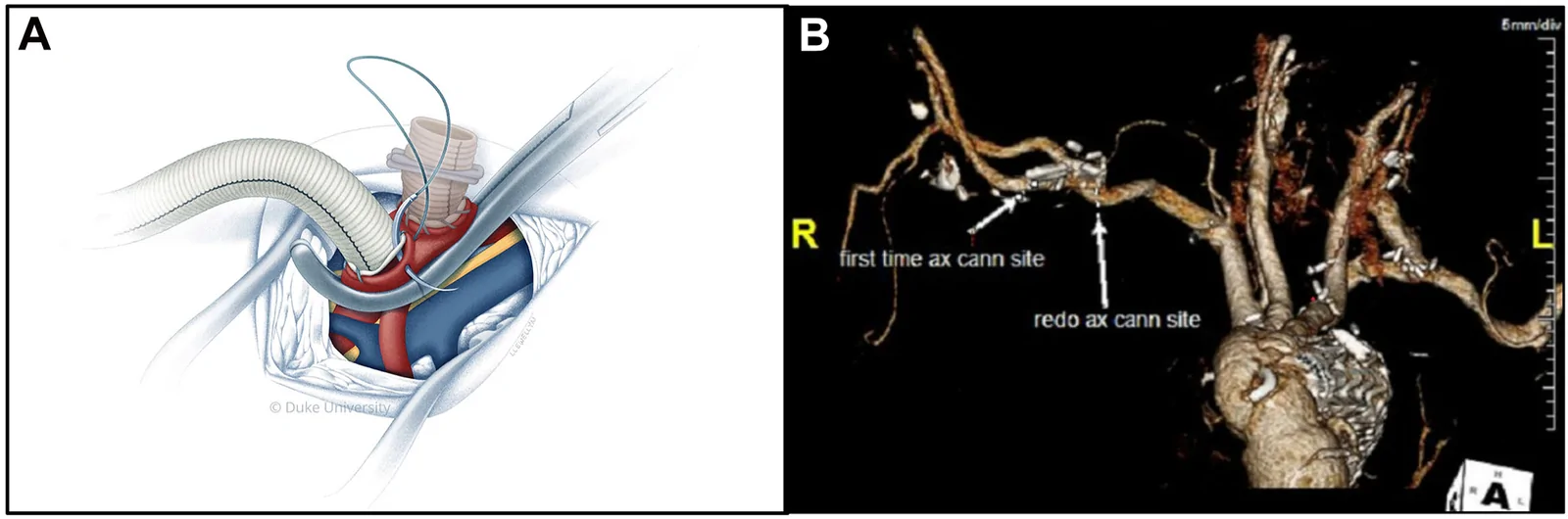
(A) Redo cannulation distal to a prior graft, demonstrating the selection of a new site on the native axillary artery when the original site is less favorable. (B) Postoperative computed tomography angiography 3-dimensional reconstruction image from a patient who underwent redo cannulation proximal to a prior graft, with the original and redo sites along the artery indicated. Note the visible clips where the prior graft stumps were clipped and oversewn. (ax cann, axillary cannulation; L, left; R, right.)

The prior incision is typically reused to optimize cosmesis and to reduce the risk of skin necrosis or wound issues that may occur with a separate parallel incision (Video).^7^ After entering through the skin and subcutaneous tissue superficial to the pectoralis major, dissection proceeds in the deltopectoral groove down to the level of the axillary artery using low-energy (20 W) cutting-current electrocautery (Figure 1A). As noted before, residual clips or graft material on the artery can serve as useful landmarks, with their location repeatedly palpated to guide dissection (Figure 1B). The artery is then mobilized circumferentially both proximally and distally to allow clamping. If a side-branch graft was used at the first operation, the brachial plexus will often be densely adherent to the graft stump and must be carefully dissected free, again with low-energy electrocautery. Depending on the location of the prior graft or direct cannulation site and the availability of an adequate arterial segment, a new side-graft anastomosis is performed at, proximal to, or distal to the original site. Once the artery has been fully mobilized and the new site chosen, 4000 to 5000 units of unfractionated heparin are administered (adjusted for patient size and anticipated extent of bleeding with sternal reentry), and the artery is then clamped proximally and distally, most often with profunda clamps. When the old side-branch site is being reused, the graft stump is trimmed to remove nearly the entirety of the stump, leaving only 1 to 2 mm attached to the artery; any thrombus within the graft stump is removed and the artery flushed with heparinized saline. A new 8-mm knitted Dacron graft is then anastomosed end-to-side to the prior site with running 5-0 polypropylene, with the bites of the suture line incorporating both the remaining Dacron graft stump and approximately 1 to 2 mm of native artery below the stump to ensure hemostasis (Figure 2). If the prior graft was placed more medially than our preferred location (a common finding in patients previously operated on at outside institutions), a site distal to the prior anastomosis is typically chosen, and the graft is sewn in the same fashion as in a primary operation (Figure 3A). Less commonly, if the prior graft was positioned more laterally, the same principles are applied (Figure 3B). In this scenario, the preexisting graft stump is left intact to avoid dislodging any thrombus that may have accumulated in the blind end. Once the patient has been weaned off CPB and the case is complete, 3 large hemoclips are placed across the base of the new graft, just above the anastomosis, to prevent the creation of a large Dacron stump that could serve as a nidus for thrombus formation.^8^ The graft is then divided just above the clips, and the stump is then additionally oversewn with running 3-0 polypropylene for secure hemostasis (Figure 3B). The soft tissues and skin are then closed in layers.

### Clinical Outcomes

Between 2008 and 2025, 55 patients (mean age, 58.9 ± 12.0 years; 72.7% male) underwent redo axillary cannulation at our institution. Most patients underwent their index operation for aortic dissection (49.1%) or aneurysm (43.6%), and the most common indications for reoperation using redo axillary cannulation were aneurysm (60.0%) and structural valve degeneration (23.6%; Table). Reoperation occurred at a median of 4.6 years after the initial operation. Repeated anastomoses were performed at the prior site in 80.0%, distal in 12.7%, and proximal in 7.3%. Thirty-day/in-hospital mortality was 1.8% (n = 1), and stroke occurred in 2 patients (3.6%). Rates of ipsilateral upper extremity neurovascular complications were 3.6% (n = 2), with no wound complications. One patient sustained an intraoperative injury to a small sensory branch of the brachial plexus, which was repaired intraoperatively and did not result in any neurologic deficits. Another patient required thrombectomy on the day of operation for brachial, radial, and ulnar artery thrombosis.

**Table.** Baseline Characteristics and Outcomes of Patients Undergoing Redo Axillary Artery Cannulation

Variable	Redo Axillary Cannulation (N = 55)
Age, y	58.9 (12.0)
Male	40 (72.7)
Indication for primary operation	
Aortic dissection	27 (49.1)
Aortic aneurysm	24 (43.6)
Infection	2 (3.6)
Other	2 (3.6)
Time from primary to redo cannulation, y	4.64 (1.95-9.02)
Primary operation at outside hospital	10 (18.2)
Indication for reoperation	
Aortic dissection	2 (3.6)
Aortic aneurysm	33 (60.0)
Aortic pseudoaneurysm	4 (7.3)
Structural valve degeneration	13 (23.6)
Infection	3 (5.5)
Site of redo anastomosis	
Initial site	44 (80.0)
Distal	7 (12.7)
Proximal	4 (7.3)
Upper extremity complication	2 (3.6)
Wound complication	0
30-day/in-hospital all-cause mortality	1 (1.8)
Perioperative stroke	2 (3.6)

Categorical variables are presented as number (percentage). Continuous variables are presented as mean (SD) or median (interquartile range).

## Comment

Reoperative sternotomy is common in adults with aortic disease, often necessitating alternative strategies for safe arterial access. Few prior studies have examined outcomes of redo axillary cannulation in patients with prior axillary use. Existing reports suggest acceptable results, and our series supports that this approach should be part of the surgical armamentarium for complex reoperations.^9^

Cannulation-related complications with axillary access are uncommon, but surgeons must remain vigilant, given the proximity of major neurovascular structures, particularly in the presence of adhesions from prior axillary use.^10^ Neurovascular injury to the dominant arm can be devastating; however, in our series, the 2 arm-related complications were successfully managed without long-term deficits. The risk of stroke, including potential embolization to the ipsilateral vertebral or carotid arteries, must also be considered, although our observed stroke rate was low in this high-risk reoperative population.

In conclusion, redo axillary cannulation is a safe, feasible option for initiating CPB during reoperative sternotomy. Although technically more demanding than primary cannulation, it should be considered whenever peripheral arterial access is required or central cannulation is not feasible. Importantly, this technique provides a reliable alternative to femoral cannulation while avoiding its known retrograde embolic risks.
