# Supplementary material for: Redo Axillary Artery Cannulation in Reoperative Cardiac Surgery: Outcomes and Technique
Source: Ann Thorac Surg Short Rep. 2026 Mar 4;4(3):742–6. doi: 10.1016/j.atssr.2026.01.020 (PMC13555482; doi:10.1016/j.atssr.2026.01.020)
Supplement: Supplementary file 1 [file mmc1.docx]

**SUPPLEMENTAL VIDEO LEGEND**

**Supplemental Video**. This video demonstrates the operative technique for redo axillary artery dissection and anastomosis of a Dacron graft at the site of prior axillary cannulation, highlighting key technical steps.
